# Relationship between *Helicobacter pylori* infection and nonalcoholic fatty liver disease: What should we expect from a meta-analysis?

**DOI:** 10.1097/MD.0000000000026706

**Published:** 2021-08-06

**Authors:** Lin Wei, Hui-Guo Ding

**Affiliations:** Department of Gastroenterology and Hepatology, Beijing You’an Hospital Affiliated with Capital Medical University, Beijing, China.

**Keywords:** *Helicobacter pylori*, meta-analysis, nonalcoholic fatty liver disease

## Abstract

**Background:**

The relationship between *Helicobacter pylori* (*H. pylori*) infection and nonalcoholic fatty liver disease (NAFLD) is a matter of debate. Although it has been studied in many observational studies, the results remain controversial. Therefore, we performed a meta-analysis to assess the association between *H pylori* infection and risk of NAFLD.

**Methods:**

We searched Pubmed, EMBASE, and Web of Science databases, from inception to September 10, 2020. Odds ratio (OR) and 95% confidence interval (CI) were pooled by random-effects model. The statistical heterogeneity among studies (I^2^-index), subgroup analyses, regression analyses, sensitivity analysis and the possibility of publication bias were assessed.

**Results:**

A total of seventeen studies involving 91,958 individuals were included in our meta-analysis. Meta-analysis of data from cross-sectional and case-control studies showed that *H pylori* infection was associated with increased risk of prevalent NAFLD (n = 15; involving 74,561 middle-aged individuals; OR1.38, 95% CI 1.23–1.55, I^2^ = 86.8%, *P < *.001). The results of meta-regression implicated that the study type and the case-control ratio impacted the total effect size. Funnel plot did not show significant publication bias. Meta-analysis of data from longitudinal studies showed that *H pylori* infection was also associated with increased NAFLD incidence (n = 2; involving 17397 individuals; OR 1.21, 95% CI 1.01–1.44, I^2^ = 6.5%, *P* = .301).

**Conclusions:**

The results indicated that a positive association between *H pylori* infection and the risk of NAFLD. Further studies are required to strengthen the association and clarify the mechanism.

## 1 Introduction

Nonalcoholic fatty liver disease (NAFLD) is characterized by hepatic steatosis while excluding alcohol use and other causes of liver diseases which is the leading cause of chronic liver diseases in the world.^[1–3]^ The prevalence of NAFLD has been estimated to be nearly 30.45% in South America, 27.37% in Asia, 24.13% in North America, 23.71% in Europe.^[4,5]^ However, the exact pathogenesis of NAFLD is still not completely clear currently, no efficient pharmacological therapy is approved for NAFLD or nonalcoholic fatty hepatitis (NASH). The human gastrointestinal tract is an integrated ecosystem, known as microbiota. Mucosal epithelium of the small intestine is a barrier between gut lumen and microbiota. Intestinal microflora is the primary source of endotoxins produced by Gram-negative bacteria (lipopolysaccharide), that normally cross the mucosa in trace amounts, enter the portal circulation and finally clear to liver cells. The increased intestinal permeability appears to be caused by disruption of the intercellular tight junctions. *Helicobacter pylori* (*H. pylori*) is a Gram-negative, spiral-shaped bacterium that colonizes the gastric epithelium. More than half of the world's population is infected with *H pylori*.^[6]^ Recent studies have shown that, *H pylori* infection plays a potential role in insulin resistance and increased intestinal permeability, which may contribute to the development of NAFLD. These studies have prompted our interest in updating current evidence. Hence, we performed an updated meta-analysis of all available evidence published to date regarding the relationship between *H pylori* infection and NAFLD.

## 2 Materials and methods

### 2.1 Registration of review protocol

This meta-analysis was conducted following the Preferred Reporting Items for Systematic Reviews and Meta-Analyses guidelines, and the protocol for this meta-analysis is available on international prospective register of systematic reviews (PROSERO; Registration number CRD42020172578).

### 2.2 Data sources and search strategy

Pubmed, EMBASE, and Web of Science databases were electronically searched from inception to September 10,2020, without language restrictions. The search terms were as follows:(“*Helicobacter pylori*” OR “*campylobacter pylori*” OR “*H pylori*” OR “HP” OR “Helicobacter spp” OR “*H. pylori*”) AND (“nonalcoholic Fatty Liver Disease” OR” Nonalcoholic Fatty Liver Disease” OR” NAFLD” OR” fatty Liver, Nonalcoholic” OR “fatty Livers, Nonalcoholic” OR “liver, nonalcoholic fatty” OR “livers, nonalcoholic fatty” OR “nonalcoholic fatty liver” OR” nonalcoholic fatty livers” OR “nonalcoholic steatohepatitis” OR “nonalcoholic steatohepatitis” OR “ steatohepatitis, nonalcoholic” OR “ steatohepatitis, nonalcoholic” OR “ NASH” OR “NAFL” OR “ fatty liver” OR” nonalcoholic fatty liver” OR” non-alcoholic fatty liver disease”). Both Medical Subject Heading and free word were used. We also reviewed references from relevant original papers and review articles to identify further eligible studies not covered by the original database research. This review was performed according to the guidelines for meta-analyses and systematic reviews of observational studies.

### 2.3 Inclusion and exclusion criteria

Inclusion criteria were as follows:

1. cross-sectional, case-control or longitudinal studies published as original articles that explored the association between *H pylori* infection and NAFLD;
2. odds ratios (OR), risk ratio (RR), hazard ratios (HR) with 95% confidence intervals (95% CI) should have been provided;
3. *H pylori* infection had to be confirmed at least one positive test as follows: serological testing (using *H pylori* IgG enzyme-linked immunosorbent assays), 13C-labeled or 14C-labeled urea breath test or fecal antigen test.
4. NAFLD had to be diagnosed by histology, imaging (mostly ultrasonography) or surrogate markers of NAFLD, such as either the hepatic steatosis index (HIS) or the NAFLD liver fat score (NAFLD-LFS), which include in their equations serum transaminase levels, body mass index (BMI), fasting insulin levels, pre-existing diabetes or metabolic syndrome.
5. all the studies included a control group.

Criteria for exclusion were as follows:

1. letters, abstracts, case reports, animal studies, editorials, reviews and meta-analyses;
2. irrelevant literatures and duplicate studies;
3. studies which did not exclude individuals with significant alcohol intake and other secondary causes of chronic liver disease;
4. studies that did not specifically report any OR or HR and 95% confidence intervals (CI) for the outcome measure of interest.

Two investigators (Lin Wei and Hui-Guo Ding) independently screened the titles and abstracts of all studies identified using the previously described search criteria to identify studies meeting the inclusion criteria. Each study meeting the requirements of the inclusion criteria then underwent a full-text independent review by both investigators. Disagreements about the inclusion of studies between investigators were resolved by discussion.

### 2.4 Data extraction and quality assessment

We extracted the following data from each study:

1. study characteristics, including the name of the first author, publication year, country of publication, study design, sample size, mean age;
2. the number of positive/negative *H pylori* infections in the NAFLD group, method of detection of both NAFLD and *H pylori* infection, the number of positive/negative *H pylori* infections in the control group, case/control ratio;
3. list of covariates adjusted in multivariable regression analyses.

We assessed the quality of each study according to the Agency for Healthcare Research and Quality (AHRQ) and the Newcastle–Ottawa Quality Assessment Scale (NOS).

### 2.5 Data synthesis and analysis

We used STATA version 12.0 software (Stata Corporation, College Station, TX) to perform meta-analyses. Odds ratio (OR) with 95% confidence interval (95%CI) was pooled by this software, which was used to describe the ratio of *H pylori* infection occurring in NAFLD patients versus the controls. Heterogeneity was assessed by Chi-square-based Q test and I^2^ -index were used to evaluate the statistical heterogeneity between the studies. The significance for the Q test was defined as *P*-value < .1. Heterogeneity was classified as follows: a I^2^ value of 0–25% indicated no heterogeneity, 26–50% indicated low heterogeneity, 51–75% indicated moderate heterogeneity, and 76–100% indicated high heterogeneity. a fixed effect model was used when I^2^ values of < 50%, and a random effect model was used when I^2^ values of > 50%.^[7]^ The subgroup analysis and regression analysis were performed to explore sources of heterogeneity. The forest plot assessed the relationship between *H pylori* infection and NAFLD. The funnel plot and Begg's and Egger's tests were used to investigate publication bias, *P* < .05 was considered statistically significant. What's more, we used the trim-and-fill method to further examine the possibility of publication bias.

## 3 Results

### 3.1 Data search and study characteristics

**Figure 1:**
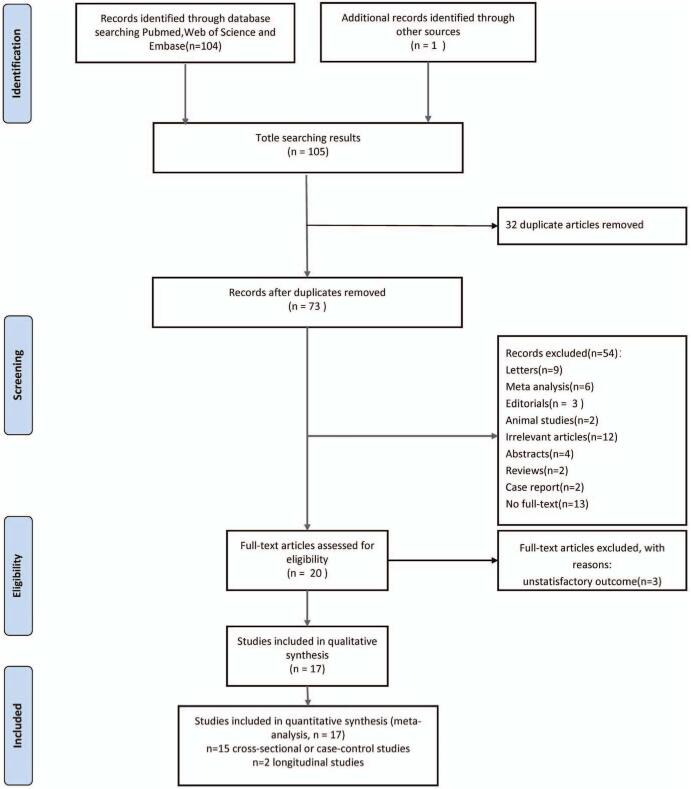
A flow diagram of the literature search and PRISMA.

The initial database search identified 104 records. Another record was identified from relevant studies. After removing 32 duplicates, 73 records remained. Of these 73 studies, 56 were excluded. Thus, 17 studies were included in the meta-analysis. A flow diagram of the literature search and the PRISMA flow diagram of the meta-analysis is shown in Fig. 1.

The main characteristics of the 17 studies are summarized in Table 1. A total of 17 studies involving 91,958 individuals were included in our meta-analysis. These studies were published between 2013 and 2020. Our meta-analysis contained 14 cross-sectional studies,^[8–21]^ 1 case-control study,^[22]^ and 2 longitudinal studies.^[23,24]^ 11 of these studies were from Asia,^[8–13,15,16,18,22,24]^ 2 from the United States,^[21,25]^ 1 from Greece,^[17]^ 2 from Egypt,^[19,23]^ and 1 from Switzerland.^[20]^

**Table 1:** Main characteristics of the 17 studies.

			NAFLD group		Control group			
Study and year	country	Study type	Hp+	Hp-	Hp+	Hp-	Diagnosis of *H pylori*	Diagnosis of NAFLD
Lu et al 2018^[8]^	China	Cross-sectional	199	397	390	881	13C urea breath test: 589 (31.5%) positive cases	Ultrasonography: 31.9% (n = 596) had NAFLD
Polyzos et al 2013^[17]^	Greece	Cross-sectional	26	2	14	11	Antibodies (IgG enzyme-linked immunosorbent assay); or 13C urea breath test: 37 (69.8%) seropositive cases	Biopsy: 52.8%(n = 28) had NAFLD
Kim et al 2017^[21]^	Korea	Longitudinal cohort study	2080	1301	7838	5809	Antibodies (IgG enzyme-linked immunosorbent assay): 9918 (58.2%) seropositive cases	Ultrasonography: 3381 individuals developed incident NAFLD on ultrasound over follow-up
Abdei-Razik et al 2018^[20]^	Egypt	Longitudinal cohort study	14	9	157	189	Faecal antigen test: 171 (46.3%) positive cases	Hepatic steatosis index (HSI) > 36 or NAFLD liver fat score (NAFLD-LFS) > −0.64; 23 individuals developed incident NAFLD over 2-year follow-up
Kang et al 2018^[14]^	USA	Cross-sectional	658	1065	1115	2566	Antibodies (IgG enzyme-linked immunosorbent assay plus anti-cagA IgG: 2655 (49.1%) seropositive cases	Ultrasonography: 31.9%(n = 1633) had NAFLD
Chen et al 2017^[11]^	China	Cross-sectional	313	290	723	937	13C urea breath test:1036 (45.8%) positive cases	Ultrasonography: 26.6%(n = 603) had NAFLD
Zhang et al 2016^[19]^	China	Case-control	300	300	144	456	14C urea breath test;444 (37%) positive cases	Ultrasonography: 50%(n = 600) had NAFLD
Baeg et al 2016^[9]^	Korea	Cross-sectional	505	440	1131	1587	14C urea breath test;1636 (44.7%) positive cases	Hepatic steatosis index (HSI) > 36 or NAFLD liver fat score (NAFLD-LFS) > −0.64 (23.3%had NAFLD)
Cai et al 2018^[10]^	China	Cross-sectional	145	288	500	1118	13C urea breath test;645 (31.4%) positive cases	Ultrasonography: 21.1%(n = 433) had NAFLD
Okushin et al 2015^[16]^	Japan	Cross-sectional	523	1279	926	2561	Antibodies (IgG enzyme-linked immunosorbent assay);1449 (27.4%) seropositive cases	Ultrasonography: 34.1%(n = 1802) had NAFLD
Fan et al 2018^[12]^	China	Cross-sectional	3905	5769	6943	11554	14C urea breath test;10848 (38.5%) positive cases	Ultrasonography: 34.3%(n = 9674) had NAFLD
Yu Y et al 2018^[18]^	China	Cross-sectional	3132	4460	4716	8081	13C urea breath test: 7848 (38.5%) positive cases	Ultrasonography: 37.2%(n = 7592) had NAFLD
Mahyar et al 2019^[15]^	Iran	Cross-sectional	22	43	15	50	Fecal *H.pylori* antigen test and antibodies (IgG enzyme-linked immunosorbent assay): 2137 (52%)positive cases	Ultrasonography: 50%(n = 65) had NAFLD
Tianjiang et al 2019^[13]^	China	Cross-sectional	1022	842	1115	1102	14C urea breath test: 2137 (52.36%) positive cases	Ultrasonography: 45.68% (n = 1864) had NAFLD
Abd-Elsalam et al 2020^[19]^	Egypt	Cross-sectional	442	82	96	26	Antibodies (IgG enzyme-linked immunosorbent assay): 538 (83.3%) positive cases	Fibroscan: 81.1%(n = 524) had NAFLD
Exadaktylos 2020^[20]^	Switzerland	Cross-sectional	40	15	0	9	histology from gastric biopsies:15 (23.4%) positive cases	NASH Clinical Research Network scoring system, NAFLD activity score (NAS) ^[32]^ and fatty liver inhibition of progression (FLIP) were used^[29]^; 86%(n = 55) had NAFLD
Alvarez 2020^[21]^	USA	Cross-sectional	222	29	145	28	Antibodies (IgG enzyme-linked immunosorbent assay) :367 (86.6%) positive cases	Hepatic steatosis index (HSI) > 36 or NAFLD liver fat score (NAFLD-LFS) > −0.64;59.2%(n = 251) had NAFLD

**Figure 2:**
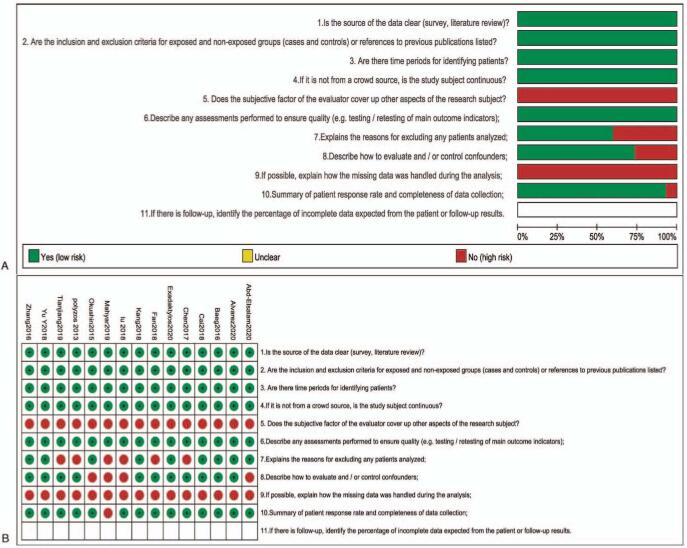
The result of quality assessment according to the Agency for Healthcare Research and Quality (AHRQ) for cross-sectional studies.

The results of quality assessment according to the Agency for Healthcare Research and Quality (AHRQ) for cross-sectional studies were presented in Fig. 2, the other 2 articles were assessed by the Newcastle–Ottawa Scale (NOS).

### 3.2 *H. pylori* infection and NAFLD prevalence

**Figure 3:**
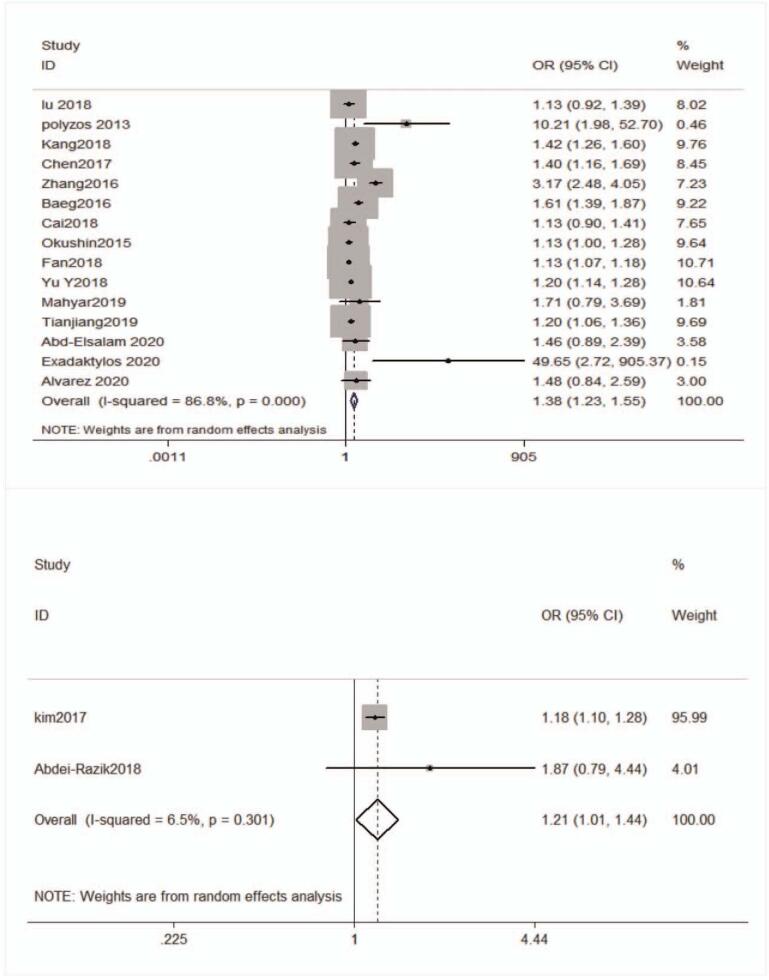
(A) The forest plot and pooled estimates of the effect of *H pylori* infection on the risk of prevalent NAFLD in cross-sectional or case-control studies. (B) The forest plot and pooled estimates of the effect of *H pylori* infection on the risk of prevalent NAFLD in longitudinal studies; CI = confidence interval, *H pylori* = *Helicobacter pylori*, NAFLD = nonalcoholic fatty liver disease, OR = odds ratio.

**Figure 4:**
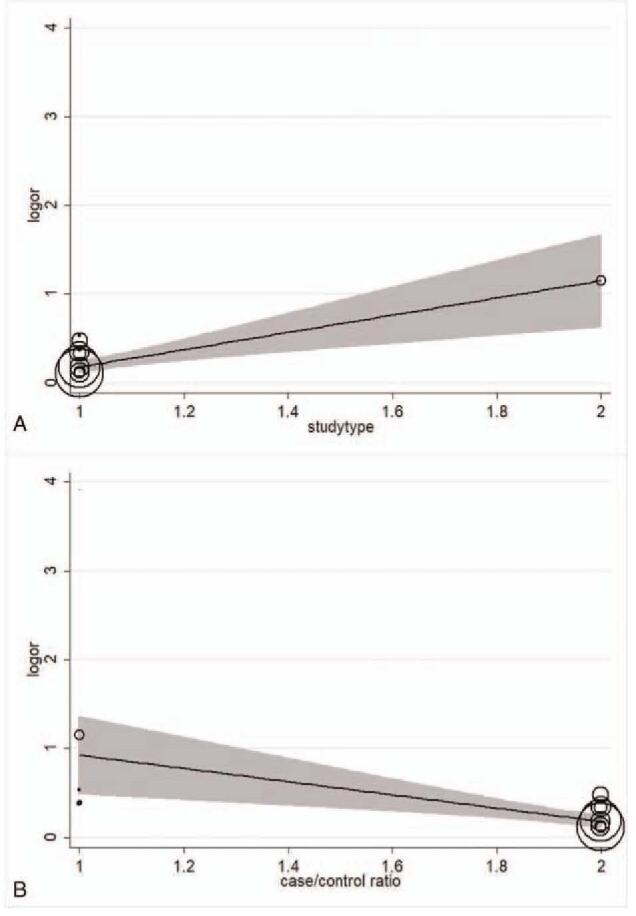
(A) The meta-regression analysis of study type. (B)The meta-regression analysis of case/control ratio.

**Figure 5:**
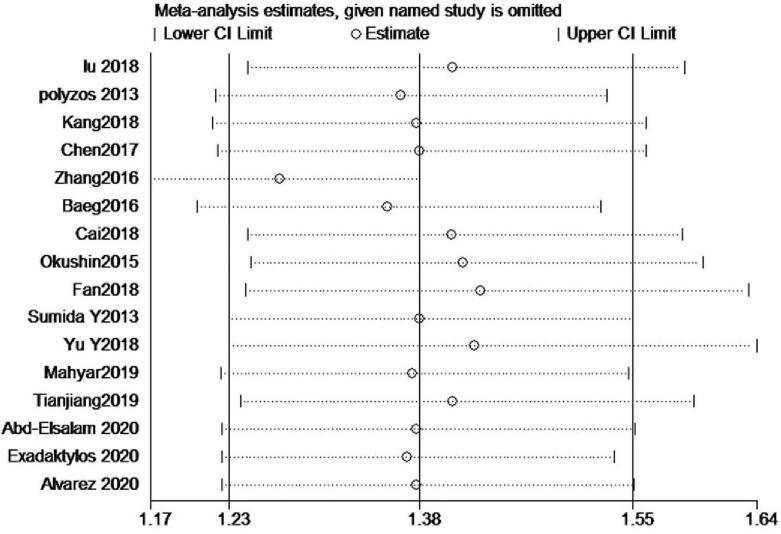
The sensitivity analysis using the one-study remove (leave-one-out) approach in order to examine the influence of each study on the overall effect size.

**Figure 6:**
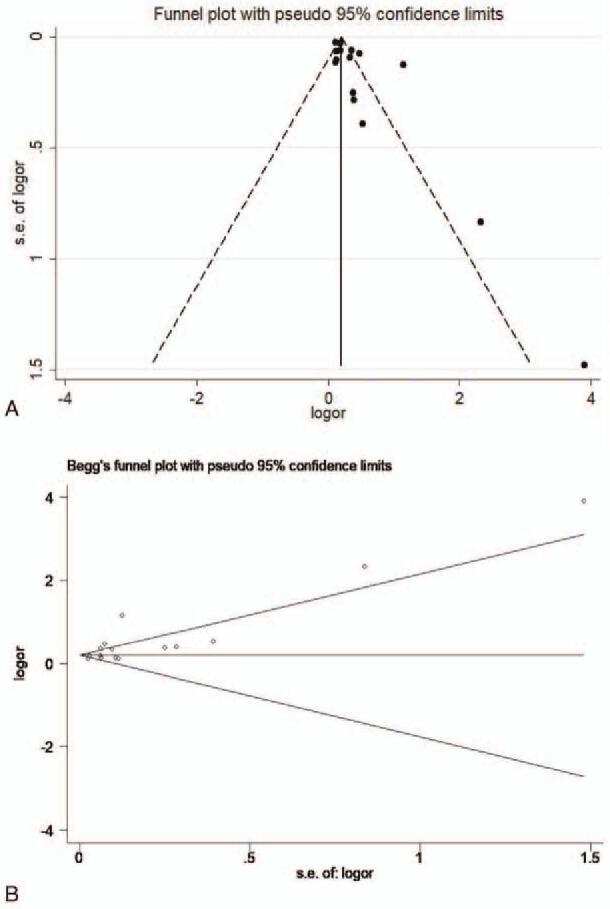
(A) Funnel plot of association between *H pylori* infection and NAFLD, (B) Begg's tests to quantify the publication bias; CI = confidence interval, NAFLD = nonalcoholic fatty liver disease, OR = odds ratio.

The forest plot and pooled estimates of the effect of *H pylori* infection on the risk of prevalent NAFLD in cross-sectional or case-control studies (involving 74,561 middle-aged individuals) were shown in Fig. 3A. The pooled overall OR of *H pylori* infection in NAFLD patients compared with the controls was 1.38 [95% confidence interval (CI): 1.23–1.55, I^2^ = 86.8%, *P < *.001]. Given the high heterogeneity, we carried out subgroup and meta-regression analyses according to the publication year, study type, the diagnosis of *H pylori* infection, the diagnosis of NAFLD, sample size, area, and case-control ratio. The result of subgroup and meta-regression analyses are shown in Table 2. Unfortunately, we did not find the cause of heterogeneity in subgroup analyses. However, the results of meta-regression implicated that the study type and the case-control ratio impacted the total effect size, the results are respectively [study type: adjusted R^2^ = 84.55%, *P* = .001,χ^2^ = 1.2%] and [adjusted R^2^ = 75.07%, *P* = .004, χ^2^ = 1.9%] (Fig. 4). We also performed a sensitivity analysis using the one-study remove (leave-one-out) approach in order to examine the influence of each study on the overall effect size (Fig. 5). The result of leave-one-out analysis implicated that the study of Zhang et al. may be a source of statistical heterogeneity, and the pooled I^2^ was 75%, and OR was 1.26 (95% CI: 1.16–1.36, I^2^ = 75%, *P < *.01; Z = 5.6 *P*_*z*_ < 0.01). We used a funnel plot to qualitatively detect the publication bias, an Egger's and Begg's tests to quantify the publication bias (Fig. 6 A/B). The funnel plots were almost symmetric. *P*-value of Egger's test was .019. As the *P*-value of Egger's test was smaller than .5, we used meta-trim command to evaluate the effect of publication bias on the results. Trimming estimator was linear and meta-analysis type was random-effects model. The result showed that no study needed to trim which means that no significant bias was observed (Fig. 6B).

**Table 2:** The result of subgroup analyses and meta-regression.

		Subgroup analysis			Meta-regression		
Stratified study	No. of studies	OR (95%CI) (Random-model)	*P*-value	I^2^ (%)	Adjusted R^2^ (%)	*P*-value	χ^2^
Year	15	1.38 (1.23,1.55)	<.01	86.8%	21.83%	.116	5.8%
>2016	11	1.24 (1.15,1.33)	<.01	59.9%			
≤2016	4	2.01 (1.22,3.32)	<.01	95%			
Study type	15	1.38 (1.23,1.55)	<.01	86.8%	84.55%	.001^∗^	1.2%
cross-sectional	14	1.27 (1.17,1.38)	<.01	72.7%			
case-control	1	3.17 (2.48,4.05)					
diagnosis of *H pylori*	15	1.38 (1.23,1.55)	<.01	86.8%	-15.67%	.703	8.7%
UBT	8	1.37 (1.19,1.57)	<.01	91.60%			
others	7	1.45 (1.13,1.85)	<.01	69.8%			
diagnosis of NAFLD	15	1.38 (1.23,1.55)	<.01	86.8%	-7.46%	.262	8.1%
ultrasound	11	1.33 (1.19,1.48)	<.01	87.3%			
others	4	2.38 (1.18,4.83)	.016	71.0%			
sample size	15	1.38 (1.23,1.55)	<.01	86.8%	-6.71%	.446	8.0%
>10,000	3	1.23 (1.11,1.36)	<.01	84.60%			
≤10,000	12	1.50 (1.23,1.83)	<.01	86.3%			
Area	15	1.38 (1.23,1.55)	<.01	86.8%	-15.93%	.397	8.7%
Asia	11	1.35 (1.20,1.52)	<.01	88.4%			
others	4	2.31 (1.11,4.79)	.01	73.4%			
case-control ratio	15	1.38 (1.23,1.55)	<.01	86.8%	75.07%	.004^∗^	1.9%
≥1	6	2.40 (1.40,4.09)	<.01	74.3%			
<1	9	1.25 (1.16,1.34)	<.01	75.60%			

The forest plot and pooled estimates of the effect of *H pylori* infection on the risk of incident NAFLD was shown in Fig. 3B, in longitudinal studies (n = 2; involving 17,397 middle-aged individuals; [OR] 1.21,95% CI 1.01–1.44, I^2^ = 6.5%, *P* = .301).

## 4 Discussion

### 4.1 Summary of evidence

Our meta-analysis includes 17 observational studies. There are 14 cross-sectional studies,1 case-control study, 2 longitudinal studies. Meta-analysis of data from cross-sectional and case-control studies has shown that *H pylori* infection was associated with the infection risk of NAFLD (n = 15; involving 74,561 middle-aged individuals; [OR]1.38, 95% CI 1.23–1.55, I^2^ = 86.8%, *P < *.001). The results of meta-regression implicated that the study type and the case-control ratio impacted the total effect size. Meta-analysis of data from longitudinal studies showed that *H pylori* infection was also associated with increased NAFLD incidence (n = 2; involving 17,397 middle-aged individuals; [OR] 1.21,95% CI 1.01–1.44, I^2^ = 6.5%, *P* = .301).

### 4.2 Comparison with previous work

Recently, Wijarnpreecha et al conducted an excellent meta-analysis and demonstrated a significantly increased risk of NAFLD among patients with *H pylori* infection (n = 6 [OR]1.21, 95% CI 1.07–1.37, I2 = 49%, *P* = .002).^[26]^ Nevertheless, the search time was from inception to June 2016, and only six studies (five cross-sectional studies and one case-control study) were included in the study. Furthermore, the previous study did not provide a detailed subgroup analysis to explore the potential influence factors in NAFLD. In addition, numerous new relevant high-quality studies on the association between *H pylori* infection and NAFLD have emerged since that meta-analysis was published. Therefore, it is necessary to implement a new meta-analysis on this issue. In the last two years, Zhou et al published a meta-analysis of 15 observational studies (eleven cross-sectional, two case-control, and two longitudinal studies) in 2019. Alessandro et al published a meta-analysis of 13 observational studies (nine cross-sectional studies including the study by Sumida et al, two case-control studies, and two longitudinal studies) in 2019.^[25]^ Compared to Zhou's and Alessandro's meta-analyses, our meta-analysis did not include congress abstracts.^[27–29]^ As article published by Sumida et al did not contain control group, our meta-analysis also did not contain their data.^[30]^ Studies in our meta-analysis almost included the articles of Zhou's and Alessandro's. What's more, we supplemented 5 new studies published in 2019 and 2020.^[13,15,19–21]^ In addition, we consulted relevant documents of the quality assessment, and we used the Agency for Healthcare Research and Quality (AHRQ) for the quality assessment of cross-sectional studies. The other two longitudinal articles were assessed by the Newcastle–Ottawa Scale (NOS).^[31]^

### 4.3 Potential explanations and implications

Firstly, NAFLD is the hepatic component of metabolic syndrome and IR (Insulin resistance) is regarded as its key pathogenetic hallmark. The accumulation of liver fat in hepatocytes caused by insulin resistance makes the liver more vulnerable to oxidative stress and subsequent lipid peroxidation. These factors lead to the development of NAFLD.^[32,33]^*H pylori* infection has been implicated in the pathogenesis of IR (insulin resistance) by many mechanisms, in particular: increased level of pro-inflammatory cytokines, eicosanoids, acute phase proteins, reactive oxygen species production, and cytokine serum changes. Many studies have demonstrated that *H pylori* infection could participate in the development of NAFLD through interfering with lipid metabolism and insulin sensitivity, stimulating and promoting oxidative stress and inflammation.^[33]^

Secondly, Hp presence has been implicated in a variety of extra-digestive conditions, in particular vascular disorders caused by the release of vasoactive and pro-inflammatory cytokines.^[34–42]^ In addition, *H pylori* presents a direct action on the hepatobiliary tract by toxins and its constituents, circulating in the portal blood^[43]^; Patients with NAFLD present a significant increase in gut permeability, and this data is positively related with liver fat accumulation. *H pylori* invasion into intestinal mucosa might increase gut permeability, resulting in more passage of endotoxins via the portal vein to the liver.^[44]^*H pylori* itself is also found in the liver and may cause inflammation and ballooning of hepatocytes.^[45]^ This *H pylori*-related hepatic injury may also play a role in the development of NAFLD.^[30]^

Thirdly, NAFLD and *H pylori* infection affect mainly aged population in the world. NAFLD is the most common liver disease in the world. A study published by Abd-Elsalam et al in 2017 showed that NAFLD was found in 42.5% of acute ischemic stroke patients. NAFLD might be associated with more severe stroke and worse outcome and the prevalence of diabetes was significantly higher among patients with NAFLD than those without NAFLD.^[46]^ On the other hand, ongoing persistence of obesity with increasing rate of diabetes will increase the prevalence of NAFLD, and as this population ages, many will develop cirrhosis and end-stage liver disease.^[47]^ a worldwide increasing prevalence of *H pylori* infection with age, reaching 40%–60% in asymptomatic elderly individuals and > 70% in elderly patients with gastroduodenal diseases.^[48]^

### 4.4 Strengths and limitations of this study

Our study has several strengths worth noting. First, we performed a rigorous search strategy and strict inclusion criteria, including all available evidence published to date. To our knowledge, ours is the largest and most updated meta-analysis to date aimed at investigating the association between *H pylori* infection and the risk of liver cirrhosis. Second, our study was registered in advance on PROSPERO platform, and most included studies were of high quality, indicating our results were reliable. Third, we conducted subgroup analyses, meta-regression and sensitivity analyses to further evaluate the association, which made our results more credible, and understood the relationship more comprehensively.

Despite these strengths, some limitations of our meta-analysis should be acknowledged. First and foremost, most of original studies are retrospective or cross-sectional design, which can only at best demonstrate an association but not causality. And the results of observational studies are generally more susceptible to bias and confounding factors than randomized studies. So further large-scale prospective studies verifying the causal relationship are needed. Second, there is a high heterogeneity in the overall results. Heterogeneity may reduce the reliability of our conclusions. However, we conducted numerous subgroup and meta-regression analyses with the hope of detecting potential factors for such heterogeneities, to find the study type and the case-control ratio may be the source of statistical heterogeneity. And most results of subgroup analyses were consistent with overall results, indicating the reliability of our results. Third, the risk factors for NAFLD include dyslipidemia, obesity, age, environment, diet and sex, and additional biochemical features of selected articles cannot be extracted, so our data integration for the meta-analysis has not been able to control these factors. Several studies did not report OR in the report OR in the results and we could not calculate adjusted OR from all the included studies. Thus, the pooled overall OR was not adjusted for confounding factors such as age, sex, BMI, and diabetes. Fourth, most of the studies were done in the Asian area, so this result was more suitable for the Asian people. Fifth, there is no uniform standard for diagnosis of NAFLD in these studies. A majority of original studies used ultrasonography and fibro-scan to diagnose NAFLD. However, diagnosis by ultrasonography has inevitable limitations due to an incorrect diagnosis of NAFLD. The last, the diagnosis of *H pylori* infection was based on serological test (ELISA), rapid urease test (RUT), urea breath test (UBT), histology or multiple means. In all of these studies, serology is the most used diagnostic method, however, its reliability is lower than other diagnostic tools such as histology or RUT.

## 5 Conclusions

Evidence from meta-analysis indicates that a positive association between *H pylori* infection and the risk of NAFLD. Further prospective studies are warranted to strengthen the association and to clarify whether there is a causative link between them. If this association is confirmed in the near future, *H pylori* infection eradication may be a new specific perspective in NAFLD therapeutic strategies.

## Author contributions

**Conceptualization:** Lin Wei, Hui-Guo Ding.

**Data curation:** Lin Wei, Hui-Guo Ding.

**Formal analysis:** Lin Wei, Hui-Guo Ding.

**Funding acquisition:** Hui-Guo Ding.

**Methodology:** Lin Wei.

**Project administration:** Lin Wei, Hui-Guo Ding.

**Writing – original draft:** Lin Wei.

**Writing – review & editing:** Hui-Guo Ding.
